# Orthodontic Bracket bonded to Enamel pretreated with Er, Cr: YSGG, silver diamine fluoride and Self-Assembling Peptide P11-4. A Lab-based, SEM Assessment

**DOI:** 10.12669/pjms.41.11.12786

**Published:** 2025-11

**Authors:** Abdullah A. Alnazeh, M. Abdullah Kamran, Abdulrahman Alshehri, Wael Awadh

**Affiliations:** 1 Abdullah A. Alnazeh Department of Pedodontics and Orthodontic Sciences, College of Dentistry, King Khalid University, Abha, Saudi Arabia; 2 M. Abdullah Kamran Department of Pediatric Dentistry and Orthodontic Sciences, College of Dentistry, King Khalid University, Abha, Saudi Arabia; 3 Abdulrahman Alshehri, Division of Orthodontics, Department of Preventive Dental Sciences, College of Dentistry, Jazan University, Jazan, Saudi Arabia; 4 Wael Awadh, Division of Orthodontics, Department of Preventive Dental Sciences, College of Dentistry, Jazan University, Jazan, Saudi Arabia

**Keywords:** Silver Diamine fluoride, Phosphoric Acid, Self-Assembling Peptides, Er, Cr: YSGG laser

## Abstract

**Objectives:**

The present study evaluates the shear bond strength (SBS) of metallic brackets bonded to enamel conditioned with Phosphoric acid (PA) and enamel conditioned with Er:Cr: YSGG laser in combination with prophylactic measures, i.e., silver diamine fluoride (SDF) and Self-assembling peptides (P114)

**Methodology:**

The present in vitro lab-based study was approved by the ethical board of King Khalid University. The study duration was three months. Total 48 maxillary premolar teeth were disinfected and categorized into four groups following different surface conditioning regimes (n=12). Group-I (Control), Group-II ECL, Group-III-ECL+SDF, and Group-IV ECL+P114. Following the enamel conditioning with different conditioning regimes, brackets were bonded, and all samples were thermally aged. A pair of bonded samples from each conditioned group was assessed for enamel bracket interface via Scanning Electron Microscopy (SEM). The remaining ten bonded samples were evaluated for SBS testing and failure analysis. Intergroup comparisons between the investigated groups were performed using one-way analysis of variance (ANOVA) and Tukey’s post-hoc test.

**Results:**

Enamel pretreated with PA demonstrated SBS values significantly higher than Group-II, Enamel conditioned with ECL only (p<0.05) but lower than Group-III- enamel conditioned with ECL+SDF and Group-IV, ECL+P114 (p<0.05)

**Conclusion:**

ECL laser combined with 38% SDF or with P114, exhibits SBS values that are notably superior to those achieved with PA.

## INTRODUCTION

While orthodontic treatments are intended to enhance the appearance and functionality of teeth, they inadvertently increase the likelihood of developing white spot lesions (WSL), also known as enamel demineralization, which poses a significant challenge for orthodontists.^1^ These lesions occur due to the prolonged presence of plaque and acid production around orthodontic appliances.^2^ Preventive measures require a comprehensive approach, including rigorous oral hygiene, professional care, and the use of remineralizing agents such as silver diamine fluoride (SDF), Casein phosphopeptide amorphous calcium phosphate (CPP-ACP), and Self-Assembling Peptide (P11-4).^3^,^4^

The use of lasers in dentistry has emerged as a promising adjunct to conventional caries prevention and treatment strategies. Laser irradiation has been investigated for its potential to enhance enamel resistance to acid attack and promote remineralization.^5^ The mechanisms by which lasers influence enamel demineralization are multifaceted and involve photothermal, photochemical, and photomechanical processes.^6^ Evidence suggests laser irradiation can enhance the uptake of fluoride by enamel, leading to the formation of fluorapatite, a more acid-resistant mineral phase. Among different lasers, the Er, Cr: YSGG laser (ECL) working at a wavelength of 2780 nm, is highly absorbed by water and hydroxyapatite, the main mineral component of enamel.^7^ Utilizing ECL as an alternative to Phosphoric acid (PA) before bonding brackets on enamel has been demonstrated to enhance the bond strength of brackets by expanding the surface area for bonding and boosting the micromechanical retention of the adhesive.^8^

Silver diamine fluoride (SDF) has gained recognition as an effective solution for halting dental caries, especially among communities with restricted access to dental care. Its mechanism of action involves the formation of silver phosphate and calcium fluoride on the enamel surface, which inhibits demineralization and promotes remineralization.^9^ The integration of ECL with SDF presents a novel approach to enhance its efficacy in reducing WSL in orthodontic patients. Similarly, Self-assembling peptides represent a promising avenue for enhancing enamel remineralization and reducing WSL due to their ability to mimic the natural enamel matrix and facilitate mineral deposition within the lesion.

This biomimetic approach to remineralization closely resembles the natural tooth formation process, potentially leading to more effective and structurally sound enamel repair.^10^,^11^ Nevertheless, the combined impact of ECL with SDF and P114 on the shear bond strength (SBS) of metal brackets attached to enamel has not been investigated and warrants further study. Based on the available indexed literature, the present study evaluates the SBS of metallic brackets bonded to enamel conditioned with PA and enamel conditioned with ECL in combination with prophylactic measures, i.e., SDF and Self-assembling peptides. It is hypothesized that ECL with SDF and self-assembling peptides (P114) will demonstrate SBS better than enamel conditioned with PA.

## METHODOLOGY

The present study followed the checklist for reporting an in vitro study (CRIS) and was approved by the ethical board of King Khalid University, IRB# KKU/COD/ETH 2024-2025/44; Dated: March 26, 2024. The study duration was three months. Total 48 maxillary premolar teeth, already extracted due to periodontal and orthodontic reasons, were selected. The teeth were cleaned with pumice (Shijiazhuang Deyoute New Material Technology Co., Ltd, Shijiazhuang, Hebei, China) following their storage in a saline-thymol solution (Anhui Shinecare Co., Ltd., Hefei, Anhui, China) for 45 days at 4°C. The inclusion criteria for teeth selection follow: teeth free from carious lesions, restorations, bleaching, stains, and anatomical anomalies. The roots of the included samples were sectioned, and the buccal surfaces were polished (UM-LZ-4, Tangshan UMG Medical Instrument Co., Ltd., Tangshan, China) using a diamond paste. All samples were randomly allocated into four groups following different surface conditioning regimes.

### Conditioning of Samples:

### Group-I (Control):

The samples in this group were conditioned with conventional 37% PA (Meta Etchant, Meta BioMed, Shanghai, China). The 37% PA gel was brushed on the buccal surface for 30 seconds, followed by an oil-free rinse of 5 seconds. Following conditioning with PA, the samples were air-dried for five seconds.

### Group-II:

Samples were conditioned with ECL (WaterLase iPlusTM, Biolase Inc., USA) irradiation adjusted at a power output of 2W and wavelength of 2.78 μm, frequency of 20Hertz and pulse duration of 140μs. The MZ6 fiber tip with a tip diameter of 0.35μm was positioned on the enamel surface perpendicularly, with a constant distance of 1 mm and a scanning speed of 2 mm/sec, both in horizontal and vertical directions, for a total of 60 seconds (30 seconds each).^12^

### Group-III:

In this cohort, samples were irradiated with ECL similar to Group-II. The irradiated samples were then smeared with 38% SDF for 60 seconds.^13^

### Group-IV:

The samples in the group were irradiated with ECL, similar to groups Two and Three. This was followed by smearing the buccal surface with Curodont™ Repair (Self-assembling Peptide P114) according to the recommendations of the manufacturer. The P-114 peptide was applied to the buccal surface of all irradiated teeth for five minutes.^14^

### Bracket Bonding and Thermal Aging:

The buccal surface of all conditioned samples was primed with a thin coat of primer (Transbond XT, 3MUnitek, St Paul, MN) for 10 seconds. Stainless steel brackets for maxillary molars with a slot size of 0.020 x 0.022 inches were adhered to the FA point located at the center of the clinical crown in orientation to the crown’s long axis via Transbond XT Light-Cure Adhesive. Using a dental explorer, the excess composite was removed around the bracket base, followed by light curing for 10 seconds each from the occlusal, gingival, distal and mesial aspects. Bracket bonding was followed by thermal aging of the samples. All samples were aged for 10000 cycles between 5°C and 60°C in a thermocycler (Infitek, Jinan, Shandon) with a dwell time of 30 sec.

### Bracket-Adhesive Interface Evaluation via SEM and SBS and SBS Assessment:

A pair of bonded samples from each conditioned group was cut cross-sectionally using the Isomet machine (Buehler, Leinfelden-Echterdingen, Germany) to assess the bracket adhesive interface via Scanning Electron Microscope (SEM) (JSM-I210, Jeol, Tokyo, Japan). The remaining ten samples were assessed for SBS testing via a universal testing machine (UTM, JC-Test510, Laizhou City, Shandong, China). The samples were mounted on the metallic stage of the UTM. Occlusal-gingival force at 1mm/min was applied to debond the metallic bracket. The force used for the debonding of the bracket was measured in megapascals (MPa). The debonded surfaces were assessed quantitatively via adhesive Remanent index (ARI). The quantitative assessment was performed at 40x magnification using a stereomicroscope (SZX10, Olympus, Japan).

### Statistical Evaluation:

To identify data normality, Levene’s test was used. Data were evaluated using means±SD. Intergroup comparisons between the investigated groups were performed using one-way analysis of variance (ANOVA) and Tukey’s post-hoc test. The analysis was performed via SPSS-22 software from IBM (Chicago, IL).

## RESULTS

**Fig.1:**
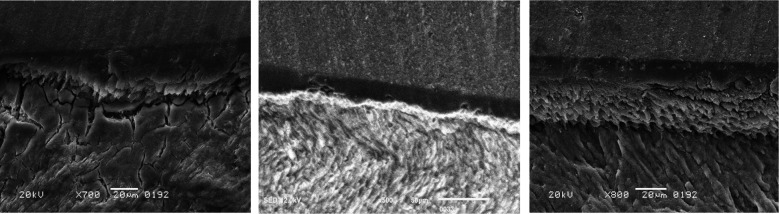
Fig.1A: Enamel Conditioned with ECL only denatured proteins and altered organic material that interferes with proper adhesive penetration. Fig.1B: Enamel conditioned with ECL and SDF. SEM image depicts remineralized enamel surface. Silver in SDF can be observed at the bracket enamel interface. Fig.1C: Enamel conditioned with ECL and P114. SEM Image shows fibrillar structures that mimic natural collagen matrices. Continuous adhesive interface between enamel and bracket.

Fig.1A. The use of ECL alone for conditioning enamel led to protein denaturation and changes in organic material, which impeded the penetration of adhesives. Fig.1B. When enamel was conditioned with both ECL and SDF, SEM images showed a surface that had undergone remineralization, with silver from SDF noticeable at the junction between the bracket and enamel. Fig.1C. Enamel treated with ECL and P114 exhibited fibrillar structures in SEM images that resembled natural collagen matrices, ensuring a seamless adhesive interface between the enamel and the bracket.

Table-I demonstrates the SBS scores of brackets bonded to enamel conditioned with different regimes. The highest bond scores were exhibited by Group-III- ECL+SD. The lowest bond scores were observed in Group-II enamel conditioned with ECL only. Enamel pretreated with PA demonstrated SBS values significantly higher than Group-II (p<0.05) but lower than Group-III- enamel conditioned with ECL+SDF and Group-IV, ECL+P114 (p<0.05). Similarly, enamel conditioned with ECL+SDF and ECL+P114 exhibited comparable bond values (p>0.05).

**Table-I:** SBS of Orthodontic Brackets Following Different Conditioning Regimes.

Investigated groups	Mean ± SD (MPa)	p-value!
Group-1: 37% PA	12.49±0.23^%^	*<0.05*
Group-2: ECL	9.16±0.11^*^	
Group-3: ECL+38% SDF	14.86±0.17^π^	
Group-4: ECL+PII4	14.28±0.82^π^	

! ANOVA Phosphuric acid (PA), Er, Cr: YSGG (ECL), Silver Diamine Flouride (SDF). Different superscript characters denote statistically significant differences. (Post Hoc Tukey Multiple Comparison Test).

**Fig.2:**
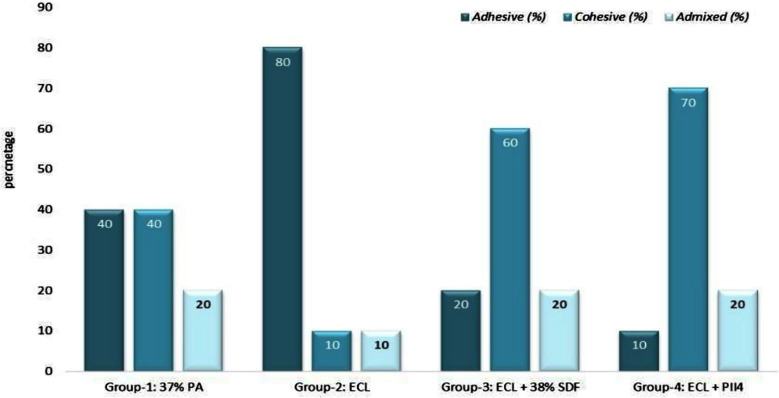
Failure assessment of debonded enamel surfaces in percentages.

The debonded surface following SBS is given in Fig.2. A balance between adhesive and cohesive failure was seen in Group-I. Whereas in Group-II majority of the failures were adhesive. In groups Three and Four, enamel pretreated with ECL+ SDF and ECL+P114 demonstrated cohesive failure type in abundance.

## DISCUSSION

The present study was constructed on the hypothesis that ECL with SDF and self-assembling peptides (P114) will demonstrate SBS better than enamel conditioned with PA. The hypothesis of the present study was accepted as enamel conditioned with ECL in combination with prophylactic measures, SDF, and P114 demonstrated bond values significantly better than control and enamel conditioned with ECL only. Better orthodontic bracket bonding significantly improves patient outcomes through several key mechanisms that affect treatment efficiency, comfort, and overall success.^15^ Superior bonding techniques reduce bracket failure rates, which directly impacts treatment timelines. Also, better bonding creates smoother surfaces around brackets with fewer retention areas for bacteria and food particles.^15^

In this study, two prophylactic agents, SDF and P114, were applied following the conditioning of enamel with ECL. It was found that the combination of ECL conditioning and SDF application had a synergistic effect on enhancing the SBS of brackets. ECL removes organic components from enamel and creates microscopic irregularities.^16^ When SDF was applied, the fluoride ions penetrated more deeply into the enamel structure through these laser-induced pathways.^17^ This increased penetration facilitated greater fluoride incorporation into the subsurface enamel layers, resulting in a more extensive fluoride-rich enamel zone. Additionally, the silver in SDF is mechanically interlocked with the laser-created surface irregularities, providing extra retention mechanisms and thus improving bond values.^18^

Similarly, when P114 was applied following ECL irradiation, the results were identical. P11-4 peptides are designed to self-assemble into fibrillar structures that mimic natural collagen matrices found in tooth development.^19^ The laser-created surface provides ideal nucleation sites for these peptides to organize and form three-dimensional scaffolds within the enamel micropores. The process created an organic framework that served as a template for subsequent mineral deposition.^20^ Further, the remineralized surface created by P11-4 treatment provides chemical compatibility with orthodontic adhesives. The newly formed hydroxyapatite crystals present optimal binding sites for phosphate-containing primers and adhesives commonly used in orthodontic bonding systems, augmenting bond strength values.^21^

Surprisingly, when ECL was used alone on the enamel surface for conditioning before bracket bonding, it deteriorated the bond strength values. Evidence suggests that ECL ablation of enamel creates irregular crater-like defects and microcracks on the surface.^22^ These structural defects create stress concentration points that weaken the bonding interface. ECL, when used as a sole conditioner, generated localized heating that denatured organic matrix components within the enamel.^23^ This thermal damage created a layer of denatured proteins and altered organic material that interferes with proper adhesive penetration and chemical bonding.^24^ SEM image supports this explanation.

Enamel conditioned with ECL+SDF and ECL+P114 demonstrated a cohesive failure type mainly. Cohesive failure within the adhesive is generally considered favorable from a clinical perspective because it preserves both the bracket and enamel integrity. Inadequate light curing or chemical polymerization, excessive adhesive thickness, and air entrapment during adhesive application create voids; all these factors can predispose to cohesive failure type.^25^ The clinical significance of these combination approaches represents a paradigm shift from the demineralization-based strategy of acid etching to enhancement-based surface modification that simultaneously improves bonding performance and enamel quality.

### Strength of the study:

This study appears to be one of the first studies to systematically combine laser conditioning with both SDF and P-114 peptides for orthodontic bonding. This is an unexplored intersection of laser technology and preventive orthodontics.

### Clinical Relevance:

The study represents a significant shift from traditional “damage-and-repair” etching to “enhance-and-protect” surface modification. This is clinically valuable because it addresses two major orthodontic challenges simultaneously - achieving strong bracket bonds while preventing white spot lesions (WSL)

### Limitations:

One of the major limitations of the present study is its in vitro design. The study does not replicate the complex oral environment that significantly influences bonding performance. Saliva composition, pH fluctuations, bacterial biofilm formation, and enzymatic activity all affect bond durability in clinical situations. Only one mechanical test, i.e., SBS, was performed in the current study. The influence of different laser types on enamel conditioning with different laser parameters, along with other prophylactic measures prior to bracket bonding, needs further evaluation and assessment. Field emission scanning electron microscopy (FESEM), along with atomic force microscopy (AFM) on enamel surface modification, needs to be explored further.

## CONCLUSION

When enamel is treated with an Er, Cr: YSGG laser combined with 38% silver diamine fluoride or with P114, it exhibits shear bond strength values that are notably superior to those achieved with phosphoric acid. The combination of Er, Cr: YSGG laser with 38% silver diamine fluoride and Er, Cr: YSGG with P114 shows promise as an alternative to using 37% phosphoric acid.

### Authors’ Contribution:

**MAK and AAlnazeh, AA, WA:** Data collection, study design, manuscript writing, manuscript drafting, data analysis and final manuscript approval.

**MAK:** is responsible and accountable for the accuracy and integrity of the present work.
